# Consensus based recommendations for diagnosis and medical management of Poland syndrome (sequence)

**DOI:** 10.1186/s13023-020-01481-x

**Published:** 2020-08-05

**Authors:** Ilaria Baldelli, Alessio Baccarani, Chiara Barone, Francesca Bedeschi, Sebastiano Bianca, Olga Calabrese, Marco Castori, Nunzio Catena, Massimo Corain, Sara Costanzo, Giacomo De Paoli Barbato, Santa De Stefano, Maria Teresa Divizia, Francesco Feletti, Matteo Formica, Mario Lando, Margherita Lerone, Fulvio Lorenzetti, Carlo Martinoli, Lorenzo Mellini, Maurizio Bruno Nava, Giuseppe Porcellini, Aldamaria Puliti, Maria Victoria Romanini, Franco Rondoni, Pierluigi Santi, Silvana Sartini, Filippo Senes, Lucia Spada, Luigi Tarani, Maura Valle, Cristina Venturino, Federico Zaottini, Michele Torre, Marco Crimi

**Affiliations:** 1 Policlinico San Martino Hospital IRCCS for Oncology, Genoa, Italy; 2 Associazione Italiana Sindrome Poland, Via Asiago, 3r, 16137 Genoa, Italy; 3 Department of Medical and Surgical Sciences for Children and Adults, University Hospital of Modena, Modena, Italy; 4 Medical Genetics, Referral Centre for Rare Genetic Diseases, ARNAS Garibaldi, Catania, Italy; 5 Clinical Genetics Unit, Fondazione IRCCS Ca’ Granda Ospedale Maggiore Policlinico, Milan, Italy; 6 Medical Genetic Unit, University Hospital of Modena, Modena, Italy; 7 Division of Medical Genetics, Fondazione IRCCS-Casa Sollievo della Sofferenza, San Giovanni Rotondo, Foggia, Italy; 8 Pediatric Orthopedic and Traumatology Unit Azienda Ospedalieria SS Antonio e Biagio e Cesare Arrigo, Alessandria, Italy; 9 Hand Surgery Unit – Azienda Ospedaliera Universitaria Integrata, Verona, Italy; 10 Pediatric Surgery Unit, V. Buzzi Children’s Hospital, Milan, Italy; 11 Azienda USL di Ferrara, Ospedale del Delta, Ferrara, Italy; 12 Medical Genetics Unit, IRCCS Istituto Giannina Gaslini, Genoa, Italy; 13 Department of Diagnostic Imaging AUSL of Romagna, U.O. of Radiology, “Santa Maria delle Croci” Civil Hospital, Ravenna, Italy; 14 Orthopedic Clinic, Department of Surgical Sciences, University of Genova, Ospedale Policlinico San Martino, Genoa, Italy; 15 Department of Muscle-Skeletal Surgery, Hand and Microsurgery Division, Modena University Hospital, Modena, Italy; 16 Plastic and Reconstructive Surgery Unit, University of Pisa, Santa Chiara Hospital, Pisa, Italy; 17 Department of Health Science, Section of Radiology, University of Genoa, Genoa, Italy; 18 Department of Morphology Surgery and Experimental Medicine, Section of Radiology, University of Ferrara, Ferrara, Italy; 19 G.Re.T.A. Group for Reconstructive and Therapeutic Advancements, University of Genoa, Genoa, Italy; 20 Department of Orthopaedics and Traumatology, Modena Policlinic, Modena, Italy; 21 DiNOGMI, University of Genova, Genoa, Italy; 22 Plastic and Reconstructive Surgery, Pediatric Surgery Unit, IRCCS Istituto Giannina Gaslini, Genoa, Italy; 23 USL Umbria 1, Ospedale Città di Castello, Perugia, Italy; 24 Hand Rehabilitation Center, Modena University Hospital, Modena, Italy; 25 Reconstructive Surgery and Hand Surgery Unit, IRCCS Istituto Giannina Gaslini, Genoa, Italy; 26 Centro di Educazione Matrimoniale e Prematrimoniale, Genoa, Italy; 27 Department of Pediatrics, “Sapienza”, University of Rome, Rome, Italy; 28 UOC Radiologia Neuroradiologia, IRCCS Istituto Giannina Gaslini, Genoa, Italy; 29 Psychiatric Service, IRCCS Istituto Giannina Gaslini, Genoa, Italy; 30 Pediatric Thoracic and Airway Surgery, IRCCS Istituto Giannina Gaslini, Genoa, Italy; 31 Kaleidos SCS-Onlus, Scientific Office, Bergamo, Italy

**Keywords:** Poland syndrome, Best practice recommendations, Diagnosis, Rare diseases, Clinical management

## Abstract

**Background:**

Poland syndrome (OMIM: 173800) is a disorder in which affected individuals are born with missing or underdeveloped muscles on one side of the body, resulting in abnormalities that can affect the chest, breast, shoulder, arm, and hand. The extent and severity of the abnormalities vary among affected individuals.

**Main body:**

The aim of this work is to provide recommendations for the diagnosis and management of people affected by Poland syndrome based on evidence from literature and experience of health professionals from different medical backgrounds who have followed for several years affected subjects. The literature search was performed in the second half of 2019. Original papers, meta-analyses, reviews, books and guidelines were reviewed and final recommendations were reached by consensus.

**Conclusion:**

Being Poland syndrome a rare syndrome most recommendations here presented are good clinical practice based on the consensus of the participant experts.

## Introduction

This project was undertaken on the behalf of the Italian Association of Poland Syndrome (AISP), a non-profit organization supporting clinical, social and scientific activities for the benefit of persons affected by Poland syndrome (PS), with the aim of providing best-practices recommendations for the management of affected individuals. This work is based on peer-reviewed scientific literature intermingled with the personal experience of the authors who are familiar with PS and come from different areas of expertise. The consensus was reached through a slightly modified Delphi process [1], which allows agreement based on guide practice when published evidence is lacking.

People living with PS usually face a diagnostic “odyssey” undergoing multiple radiological, clinical and genetic tests, which are not always suitable. Published materials and personal experiences were here discussed: the provided recommendations should be considered a consensus based on the opinion of clinical experts with years of experience on PS, as further levels of evidence are not always available in the literature. People with PS typically miss one pectoralis major muscle [2]. In most affected individuals, underdevelopment selectively affects the sternocostal head of the affected pectoralis major muscle with consequent asymmetry of the chest. In some cases, additional omolateral muscles of the chest, shoulder and upper limb, may be missing or underdeveloped. Bones of the rib cage can be also affected with shortened/missing/supranumerary ribs and fused/malformed/supranumerary vertebrae, potentially leading to more severe thoracic deformations and respiratory distress. Mammary gland and nipple hypo/aplasia also occur along with sparse or ectopic omolateral axillary hair [3].

A subset of PS patients also have hand malformations affecting the same side and commonly including brachydactyly with or without syndactyly of the central fingers [4, 5]. Additional upper limb anomalies of PS include mild shortening of forearm bones and mild underdevelopment of the arm muscles; but these features are not easily detected unless accurate measurements are carried out. In the absence of bony involvement, musculoskeletal manifestations of PS usually have trivial or minor health implications and, therefore, can go unnoticed for years. By contrast, severely affected individuals with multiple muscle and bony abnormalities of the chest, upper limb or both are recognized at birth. In a minority of the cases, PS combines with extra-musculoskeletal congenital defects such as lung or kidney malformations and dextrocardia [6]. Rarely, chest and/or upper limb abnormalities of the PS type occur bilaterally [7]. Although bilaterality might be an underestimated feature of PS, the existence of a PS variant symmetrically affecting both sides of the thorax is still a matter of debate [8].

## Main text

### Etiology and pathogenesis of PS

To date, the etiology and pathogenesis of PS are still unknown. Most PS cases are sporadic. This evidence together with the striking asymmetry characterizing the typical patients are arguments against a mendelian etiology for PS. Nevertheless, a genetic etiology cannot be completely excluded due to the existence of familial cases, as well as the possibility of genetic mosaicism potentially explaining also the sporadic ones [9, 10]. Indeed, sporadic cases could be also explnained by the intervention of genetic and environmental factors according to a multifactorial poligenic model. Concerning the pathogenesis of PS, there are many available hypotheses in literature. The most commonly agreed one is that dysembryological process leading to PS is caused by a vascular defect of the subclavian artery (which nurtures the developing scapulo-humeral girdle region) around the 45th day of embryonic life. This causes an early insufficiency of inflow to the distal limb and to the breastplate, eventually leading to the main musculoskeletal features of PS. According to this model, the vascular insult might be secondary to a variety of environmental and constitutional factors which remain undefined [11]. Alternatively, and considering PS as a polygenic trait, it may be due to the involvement of genes regulating embryonic development, in particular of pectoral girdle, whose variants could be transmitted to the patient from healthy parents and be at the origin of PS.

## Methods

### Consensus decision making to reach agreement among participants

A working group of 35 experts has been set up, including clinical and molecular geneticists, pediatricians, plastic, thoracic and hand surgeons, psychologists, radiologists and physical therapists, to formulate best practice recommendations for PS based on the consensus reached through the Delphi process, which has been recognized as a valid approach to ensure relative anonymity and economy. Three experts (MC, IB, LM and MT) have identified five main areas to be explored: 1) Diagnosis, 2) Surgical treatments and specific medical approaches on the major symptoms, 3) Plastic surgery and rehabilitation, 4) Psychological issues and social assistance, 5) Clinical follow-up and general management. Each participant was asked to provide provisional recommendations (*N* = 103) based on their personal experience and bibliographic knowledge: the PubMed/MEDLINE electronic databases were searched for suitable articles from 1990 to 2019. All PS synonyms were searched in MeSH terms, title and abstract. Subsequently, the panel members independently evaluated the initial recommendations proposed regarding the topics listed above in an online survey (100% response rate): the participants were asked in two subsequent rounds of surveys whether they agreed, disagreed (they were also asked to provide a reason) or were unable to comment on any of them. The findings resulted in three possible grade levels: i) certainly useful/strong literature, ii) possibly useful/modest literature, or iii) Good Clinical Practice (GCP) but no relevant literature available. The recommendations were accepted if ≥50% of the experts agreed. For the last step, the recommendations approved by the previous surveys were discussed in a face-to-face consensus meeting held on October 2019 and hosted in the Policlinico Hospital in Modena (number of experts participating: 21). Disagreements were resolved through discussion or asking for the opinion of a third expert. Although consensus was reached, comments were considered to better clarify the recommendations. An expert without the right to vote (MC) facilitated the entire process.

## Results

### Diagnosis

Unilateral agenesis or hypoplasia of the (sternocostal head of the) pectoralis major muscle is currently considered pathognomonic of PS (Table 1) that is still exclusively a clinical diagnosis (for the additional features, refer to the Principal diagnostic criteria section).

**Table 1:** Recommendations for diagnosis of PS (major complication)

		Grade	Consensus agreement
R1.1	Major complications are related to the severity of thoracic and upper limb defects; there are different complications, such as functional and structural limitations of compromised thoracic region and of upper limb.	Definitely useful/strong literature	86,7%
R1.2	Aesthetic problems can determine psychological serious issues of patients and their parents. Psychological issues may be of different severity according to the gender and the education level.	Definitely useful/strong literature	100%
R1.3	After the diagnosis of PS, US is indicated to exclude intra-abdominal, renal and heart structural anomalies.	Possibly useful/modest literature	100%

**Fig. 1:**
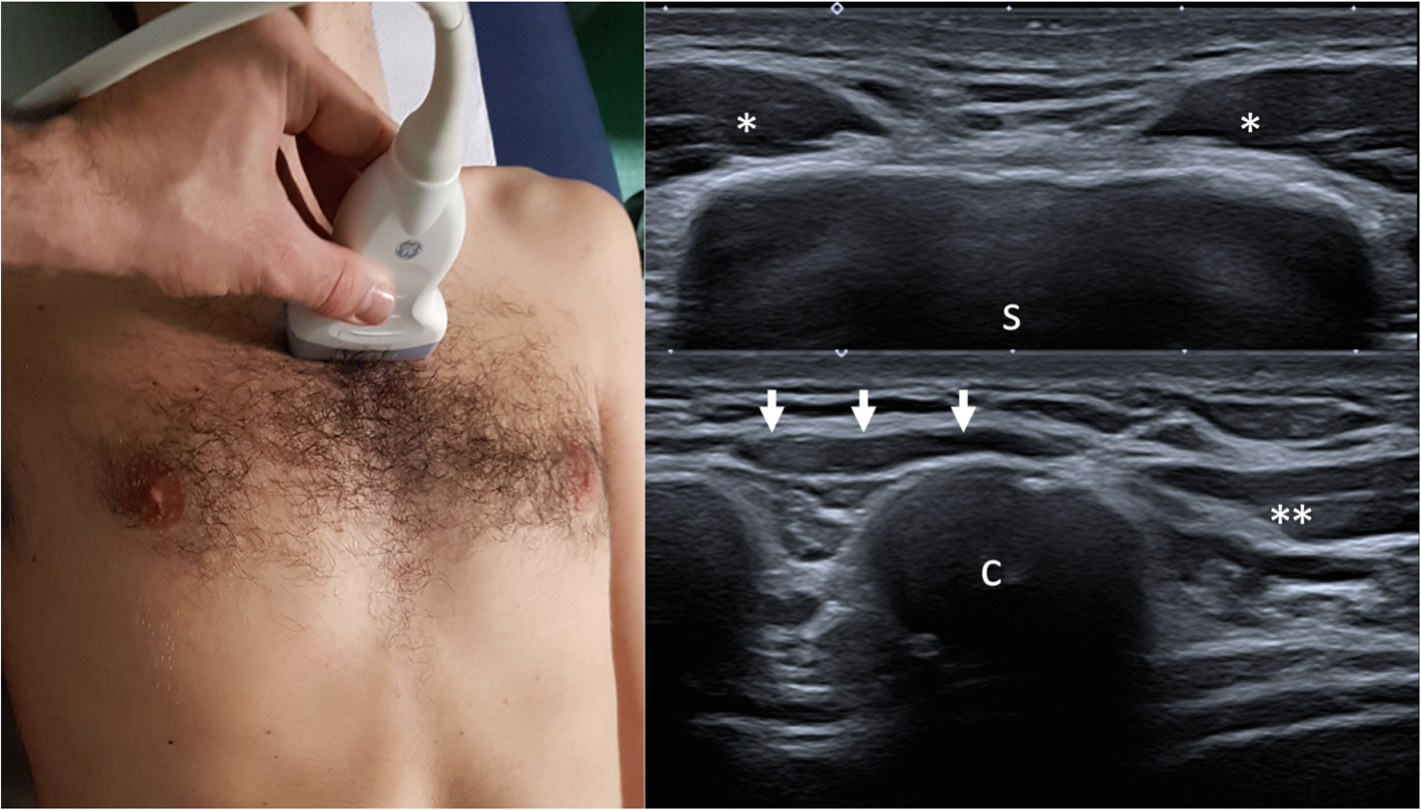
Transverse over the sternum. Probe placed transversely on the sternum (s) with the insertion of sterno-costal component of pectoralis major muscle (*) on both side of the manubrium and body of sternum, providing immediate information regarding any asymmetry of this component. In order to check the lower part of sterno-costal component is necessary moving with the probe distally to the sixth costal cartilage (c) and the first fibers of rectus abdominis (arrow)

#### Clinical presentation

The clinical diagnosis of PS may be suspected in neonatal/pediatric age in front of patients who present i) asymmetric hypoplasia of the pectoral muscles and any abnormalities of the thoracic cage, or ii) anomalies affecting the pectoral muscles unilaterally. Patients are rarely reported with bilateral pectoralis major muscle agenesis/hypoplasia. In adulthood, patients with unexplained muscle hypoplasia may be diagnosed with PS, especially if associated with asymmetric of the thoracic cage, anomalies of the mammary region (hypoplasia or agenesis of the breast and of the unilateral nipple) and abnormalities of the hand.

#### Clinical diagnosis

Since a gene(s) responsible for PS has not yet been identified, the diagnosis of PS is clinical and based on the recognition of the characteristic recurrent pattern of features along with an appropriate differential diagnosis. Agenesis or hypoplasia of the pectoralis major muscle is currently the cardinal feature mandatory for the diagnosis (Table 2). The pectoral muscle anomaly is generally easily observed by asking the patient to push the palms of the hands against each other with the arms positioned in front of the body [12]. Additional commonly reported features include:

- Absence or hypoplasia of other chest muscles: small pectoral muscle, anterior tightened muscle, gran dorsal muscle, deltoid muscle
- Anomalies of the thoracic cage: agenesis or hypoplasia of one or more costal segments, pectus carinatum, excavatum, clavicular hypoplasia, pulmonary herniation
- Abnormalities of the mammary region: agenesis or hypoplasia of the breast, areola and nipple
- Abnormalities of the upper limb and shoulder
- Absence or asymmetric reduction of axillary hairs
- Other associated skeletal anomalies: Sprengel anomaly (congenital elevation of the scapula secondary to absence of the upper portion of the serratus anterior muscle), radioulnar synostosis, emivertebre, vertebral fusions.

**Table 2:** Recommendations for diagnosis of PS (principal diagnostic criteria)

		Grade	Consensus agreement
R2.4	The mandatory feature of PS is the agenesis or hypoplasia of the pectoralis major muscle (the sterno-costal head is always affected). In most cases, PS is unilateral. Presumed bilateral PS needs a more extensive differential diagnosis. Additional diagnostic criteria are hypo/aplasia of the omolateral mammary gland and nipples, and malformations of the omolateral upper limb (limited to or more severely affecting the central rays).	Definitely useful/strong literature	93,8%
R2.5	The diagnosis is made through the physical examination of the patient; an ultrasound of the pectoralis muscles is important but not strictly necessary for the diagnosis	Definitely useful/strong literature	92,9%
R2.6	The sterno-costal head of the pectoralis major muscle is involved in most frequently; the other heads of the pectoralis major muscle and the pectoralis minor muscle are involved in different percentages of patients	Definitely useful/strong literature	93,3%
R2.7	The latissimus dorsi muscle may be involved too in a minority of cases	Definitely useful/strong literature	92,3%
R2.8	Many variable phenotypical characteristics can be associated but we cannot diagnose PS in the absence of the basic diagnostic criterion	Definitely useful/strong literature	92,9%
R2.9	The concurrence of rare internal organ malformations, such as kidney agenesis or destrocardia, may ease prenatal detection, but also in these cases, the underlying PS is recognized postnatally	Definitely useful/strong literature	71,4%

**Fig. 2:**
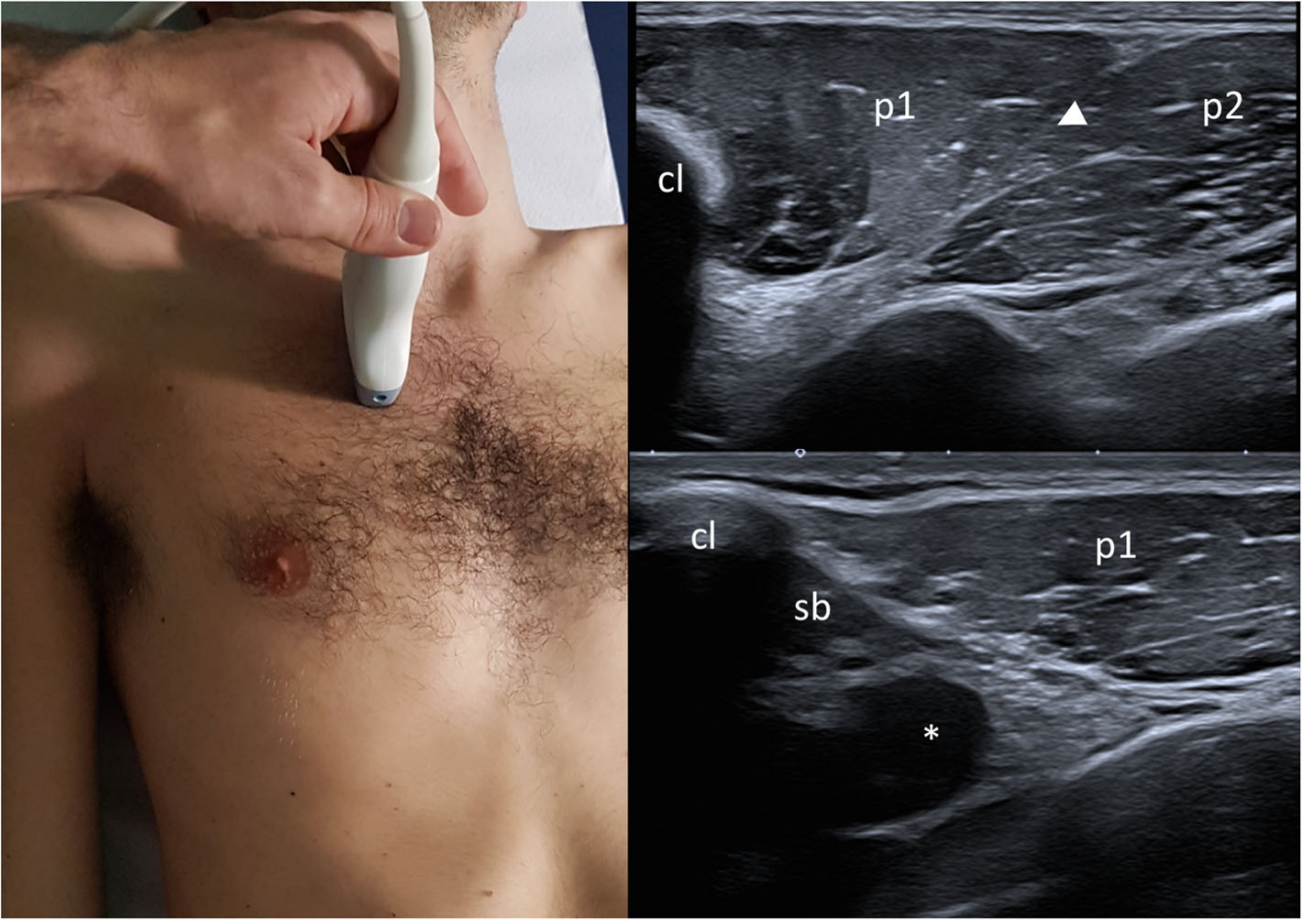
Sagittal parasternal with the upper edge of the probe on the clavicle. Probe on the sagittal axis of the clavicle, immediately lateral of sterno-clavicular joint: at the upper edge of probe there is the clavicle (cl) where clavicular component of pectoral major (p1) takes origin; a cleavage plane (arrow head) with the sterno-costal component (p2) is visible. Starting from the midportion of the clavicle and continuing laterally, the subclavius muscle (sb) is identified underneath the bone, parallel to the long axis of clavicule, crossed below by axillary artery (*) and vein (collapsed in the image) and brachial plexus cord

They are also described as associated malformations:

- genitourinary malformations
- cardiac malformations
- hepatic and/or biliary tract malformations.

#### Radiological examination

Ultrasound (US) represents the first line diagnostic imaging method to support or confirm the clinical diagnosis of PS, due to its easy accessibility, cost-effective and radiation free modality. An accurate description of soft tissue and chest wall morphological abnormalities, not always easy to identify during clinical assessment, is of outstanding importance since to different types of malformation correspond a different therapeutic approach. Evaluation by US is therefore recommended (Table 3) to assess pectoralis muscle (major and minor), breast characteristics of the two halves of the thorax, and could be used as screening exam for cardiac and renal abnormalities [13]. Associated rib abnormalities are detectable by US but, when suspected, imaging work-up should be completed with other diagnostic modalities described later. In case of specific clinical concern, by means of US performed by radiologists with expertise in musculoskeletal pathology, is possible to examine complex dysmorphism of distal upper extremities: i.e. mapping of hand muscle could address surgery and rehabilitation, moreover in pediatric patient less suitable for MRI scanning.

**Table 3:** Recommendations for radiological examination

		Grade	Consensus agreement
R3.10	Evaluation by ultrasound is recommended to assess pectoralis major, subcutaneous tissue and breast characteristics of the two halves of the thorax. US examination, because of its more availability, not radiation exposure for the patients and cost-effectiveness, should be the first line (and often the only one) imaging tool in order to confirm the clinical suspect of PS (agenesia or hypoplasia of the pectoralis major muscle) and to assess the severity of anomalies.	Definitely useful/strong literature	92,3%
R3.11	Evaluation by CT (without contrast medium) is recommended only for abnormalities of the rib cage that require surgery in non-adult patients	Possibly useful/modest literature	100%
R3.12	Pre-natal suspect of PS is sometimes possible as a collateral finding in routine US morphological examination if there are severe bone manifestations involving hands or rib cage, but it has to be confirmed with clinical examination after birth. On the basis of the current state of knowledge no further radiological analysis are indicated in uterus if parents are affected by the syndrome.	GCP (no literature available)	100%
R3.13	Radiological workup includes: ultrasound of the pectoralis region, chest X-ray, cardiac evaluation with echocardiography, abdominal US, other examinations on the basis of the specific phenotype	Definitely useful/strong literature	100%
R3.14	Ultrasound is able to categorize pectoralis major abnormalities in three classes (i.e. i, total agenesis of the muscle; ii, agenesis of the sternocostal part with normal costoclavicular component; iii, partial agenesis of the sternocostal part with normal costoclavicular component) as well as to recognize regional anomalies affecting the pectoralis minor and regional vessels	Definitely useful/strong literature	90,9%
R3.15	MR imaging has limited indications, particularly when ultrasound is non-conclusive	Possibly useful/modest literature	77,8%
R3.16	Chest radiography should be obtained routinely for gross evaluation of the rib cage and the heart	Definitely useful/strong literature	88,9%
R3.17	Imaging is not always necessary for the diagnosis of PS, however it may be helpful for the surgical planning	Definitely useful/strong literature	77,8%
R3.18	CT or MRI often more clearly depict the absence of the pectoralis major muscle and allows better appreciations of other nearby associated musculoskeletal anomalies but should not be indicated in a routine radiologic evaluation in a primary diagnostic phase	Definitely useful/strong literature	88,9%
R3.19	US examination should be performed with an high frequency probe, with a musculoskeletal preset, and should be bilateral and comparative, detecting all the 3 heads of the pectoralis major muscle (clavicular, sternocostal and abdominal) with sagittal and trasverse scans, the pectoralis minor muscle and the mammary gland	Definitely useful/strong literature	100%
R3.20	X-rays of the thorax or of the ribs are not specific for PS and not often necessary in diagnostic phase but can help showing associated malformation of the rib cage	Possibly useful/modest literature	90,0%
R3.21	Only in few selected patients should be considered a complete radiologic study of the skeletal and muscles of hands, forearms, upper arms and/or scapulas with X-rays or CT (other than MRI) in order to better detect complex anomalies clinically evident and define a pre-surgical assessment.	GCP (no literature available)	62,5%
R3.22	Chest X-Ray if there is clinical suspicion of rib agenesis. CT scan if severe deformity of the rib cage is observed; cardiac and renal US evaluations could be performed to exclude cardiac or renal anomalies.	Possibly useful/modest literature	100%
R3.23	In complex deformities of the chest wall, CT scan may provide a more detailed depiction of bone anomalies and vascular relationships. Multi-imaging evaluation may be needed in case of hand deformities to support clinical assessment.	Definitely useful/strong literature	81,8%

**Fig. 3:**
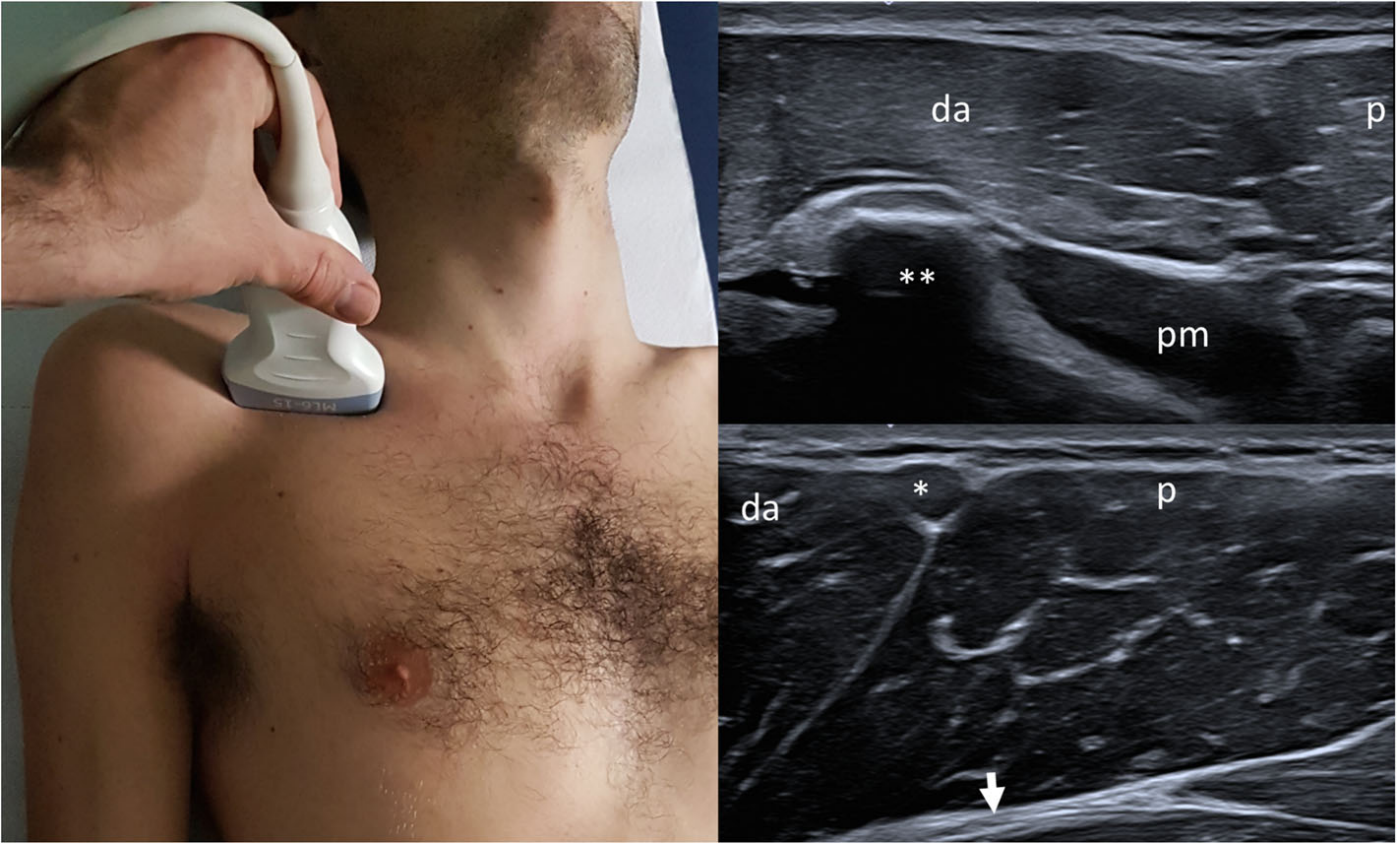
Transverse over the coracoid immediately inferior to the clavicle and medial to gleno-humeral joint. Probe placed transversely over the coracoid (**), found immediately inferior to the clavicle and medial to gleno-humeral joint; on the medial side of the coracoid is possible to appreciate the pectoralis minor muscle (pm). Superficial to the coracoid is located the anterior head of deltoid (da), to differentiate from clavicular component of pectoralis major (p) identifying the cephalic vein in between them (*): pectoralis major muscle intramuscular tendon (arrow)

**Fig. 4:**
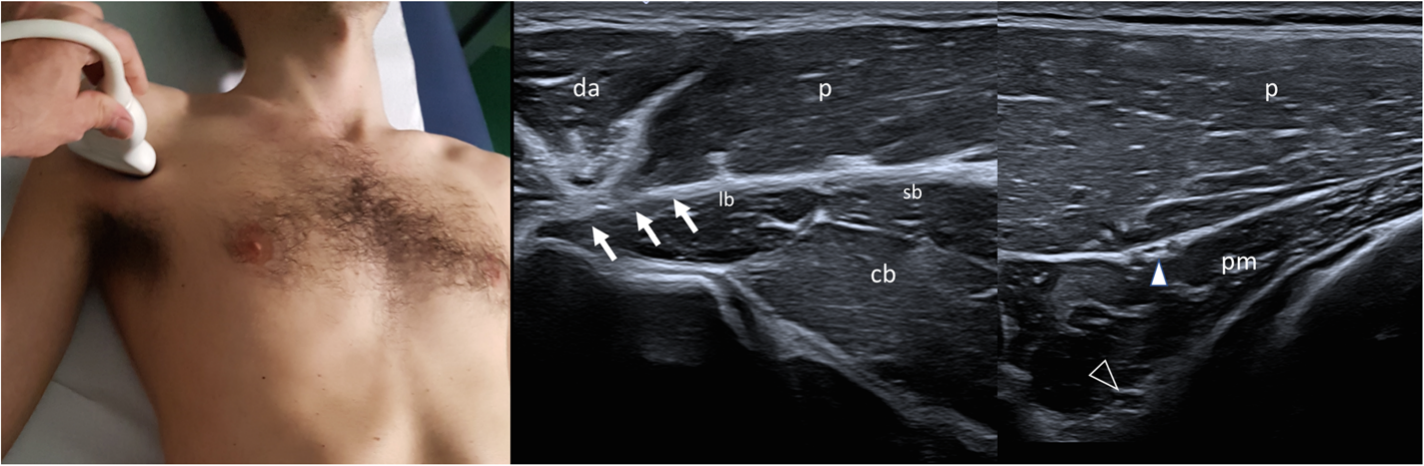
Transverse on the arm in external rotation. Probe placed transversely on the arm in external rotation: pectoralis major tendon (arrows) inserting on the lateral labrum of bicital groove; the tendon is located superficial to long head of the biceps (lb), short head of the biceps (sb) and coracobrachialis muscle (cb). Moving the probe medially keeping the same orientation, the pectoralis minor appears in between pectoralis major and thoracic wall, and two neurovascular bundles for pectoralis muscle are visualized (lateral pedicle white arrow head, medial pedicle black arrow head)

US examination should be performed with an high frequency probe, with a musculoskeletal preset, and should be bilateral and comparative, detecting all the 3 heads of the pectoralis major muscle (clavicular, sternocostal and abdominal) with sagittal and transverse scans, the pectoralis minor muscle and the mammary gland. Thereby US allows to classify different pectoralis major abnormalities into three morphologic groups: i, total agenesis of the muscle; ii, agenesis of the sterno-costal part with normal clavicular component; iii, partial agenesis of the sterno-costal part with normal costoclavicular component. Moreover US is able to recognize regional anomalies affecting the pectoralis minor and regional vessels [14]. For evaluation of pectoralis major and minor muscle, this “4 view” standard assessment is proposed (Table 4, Figs. 1,2, 3, and 4). In Prenatal setting, US screening is strongly limited by the difficulties in appreciating such a thoracic asymmetry. Only severe bone manifestations involving hands or rib cage might be identified on occasion of US morphological examination, but fetal movements often reduce the detection rate especially in low-risk pregnancies. Any prenatal findings is aspecific and need to be confirmed with clinical examination after birth [15]. On the basis of the current state of knowledge no further radiological analysis are indicated in uterus if parents are affected by the syndrome. A complete radiological workup as well as US of the pectoralis region, echocardiography and abdominal ultrasound, inckudes also X ray of the thorax if rib cage abnormalities are clinically suspected [16]. In selected case of severe chest malformation, thoracic CT and /or MRI are appropriate for a thorough pre-surgical assessment. In particular CT scan with contrast may provide a more detailed depiction of bone anomalies and vascular relationships. Only in few selected patients should be considered a complete radiologic study of the skeletal and muscles of hands, forearms, upper arms and/or scapula with X-rays or CT (other than MRI) in order to better detect complex anomalies clinically evident and define a pre-surgical planning [17, 18]. Wide consensus is reached among experts about the “second line” role of CT and MRI in PS diagnostic-therapeutic process. Both CT and MRI clearly depict the absence of the pectoralis major muscle and allows better appreciations of other nearby associated musculoskeletal and inner organs anomalies but, considering radiation exposure of CT, necessity for anesthesia in younger pediatric patient who undergoes MRI other than lower cost-effectiveness, should not be indicated as first approach. In setting of pre-surgical planning, MRI can provide the most of necessary information without exposing patient, often in young age, to the risk of radiations. Regarding thoracic MRI, in order to obtain a highly defined picture of a structure in constant movement (breathing related), specific sequences and adjustments are required.

**Table 4:** “4 view” standard assessment for evaluation of pectoralis major and minor muscle

Probe Position	Description
Transverse over the sternum	Moving in cranio-caudal direction to demonstrate the sternal component of the pectoralis major muscle over the sternocostal junctions and comparing to the opposite side in order to detect any asymmetry (Fig. 1)
Sagittal parasternal with the upper edge of the probe on the clavicle.	Moving laterally to demonstrate the clavicular component of pectoralis major. Switching color Doppler on to demonstrate the position of cephalic vein as a landmark to distinguish the clavicular component of the pectoralis major from the anterior component of deltoid (Fig. 2)
Transverse over the coracoid immediately inferior to the clavicle and medial to gleno-humeral joint	On the medial side of the coracoid is possible to appreciate the tiny tendon by which pectoralis minor muscle takes origin. Moving caudally the muscle appears between the pectoralis major superficially and rib with interposed intercostal muscle on the depth (Fig. 3)
Transverse on the arm in external rotation	Moving in cranio-caudal direction from the humeral head along the tendon of the long head of the biceps up to the myotendinous junction to demonstrate the overlying pectoralis major tendon and its insertion into the humerus (Fig. 4)

#### Differential diagnoses

Literature is scanty on the differential diagnosis of PS. Until the preparation of this manuscript, 33 papers appeared in PubMed under the research strings [“Poland syndrome” AND “differential diagnosis”] and [“Poland sequence” AND “differential diagnosis”], or [“Poland’s syndrome” AND “differential diagnosis”] and [“Poland’s sequence” AND “differential diagnosis”]; but, among them, differential diagnosis of PS was discussed only in four of them [19–22]. Of these papers, two were dedicated to the radiological differential diagnosis of unilateral hyperlucent lung at standard radiographs [20, 21], one is a systematic review on controversies in PS [22], and the remaining is a case report pointing out a possible overlap between PS and localized (thoracic) lipoatrophy [19]. Accordingly, a satisfactory differential diagnosis of PS is carried out by the intersection of practitioner’s experience on human malformation patterns and the “complexity” of the presenting phenotype. Scenarios of differential diagnosis of PS include:

- Unilateral PS without additional skeletal/limb anomalies;
- Unilateral PS with additional skeletal/limb anomalies;
- Bilateral PS;
- PS “plus” (i.e. PS with additional anomalies not regularly considered part of the PS phenotypic spectrum);
- Unilateral hyperlucent lung at thoracic X-rays.

The diagnosis of PS is usually clear-cut in the “typical” patient presenting with isolated unilateral hypo/aplasia of the major pectoris muscle with or without hypo/aplasia of the overlaying mammary gland/areola/nipple (Table 5). The most common differential diagnoses include thoracic asymmetry due to clinically significant thoracic scoliosis and/or anomalies of the bony structures (sternum, ribs, vertebrae), isolated congenital mammary gland/areola/nipple asymmetry (particularly, in women) or unilateral hypo/aplasia, and consequences of thoracic traumas/surgery. Additional disorders that may present cause localized hypo/atrophy of the thoracic soft tissues include localized lipoatrophy [19], morphea or localized scleroderma, Becker nevus syndrome and Parry-Romberg syndrome (extending to the thoracic soft tissues). Finally, thoracic asymmetry might be also the result of unilateral diaphragmatic acquired or congenital abnormalities. Physical examination accurately assessing the soft tissues of the thoracic cage in combination with scrutiny of the past medical history and, in selected cases, radiological investigations support the practitioner in the differential diagnosis. In patients presenting with (usually) unilateral or asymmetric upper limb involvement, the identification of the concurrent omolateral pectoralis anomaly, which is considered necessary and pathognomonic of PS, separates patients with PS from those with partially overlapping upper limb malformations. Considering the pattern of upper limb involvement in PS, the most common differential diagnosis is ectrodactyly, which defines a developmental deficiency of the distal central rays of the limb resulting in hypo/aplasia of the central digits extending, in some patients, to the corresponding metapods. The typical presentation of hand ectrodactyly is a two- or three-finger hand with a cleft extending up to the carpal bones. Less frequently, the typical brachysyndactyly observed in PS might be confused with the more common transverse terminal limb deficiencies. In these cases, differential diagnosis is usually resolved at physical examination of the thoracic cage. Patients with bilateral PS are rare in clinical practice, although some authors speculated that, in PS, bilateral features should not surprise and could be underestimated in the current literature [8]. In PS, bilateral presentation rises additional, though exceptional differential diagnoses mainly in the field of skeletal dysplasias leading to thoracic cage hypoplasia. Accordingly, one case with “bilateral” PS has been published, which has been subsequently reassigned to the diagnosis of “thoracic dysplasia” [23, 24]. In this scenario, physical examination by an expert in human malformation patterns and total-body skeletal radiograph allows differential diagnosis. Exceptionally, PS presents in combination with additional anomalies, which are nosologically separated from PS. Sprengel deformity (i.e. hypoplasia, and upper and medial dislocation of the scapula) has been recently recognized as the most common accompanying feature of PS [25]. PS can also associate with other (usually) sporadic malformation patterns (with unknown or heterogeneous etiology) affecting the cephalic pole and/or the upper segments of the musculoskeletal system. In particular, PS combines recurrently with Moebius syndrome in the literature and this phenotype has been defined Poland-Moebius syndrome [26]. In single reports, PS concurs with Adams-Oliver syndrome [27], Klippel-Feil syndrome [28], facio-auriculo-vertebral dysplasia [29], and frontonasal dysplasia [30]. Carey-Fineman-Ziter syndrome is an ultrarare congenital myopathy with marked facial weakness and additional features, also including PS [31]. Carey-Fineman-Ziter syndrome is easily differentiated from the “simpler” PS by the unique pattern of presenting features. It has been recently associated with recessive variants in *MYMK* [32]. Unilateral agenesis of the major pectoris muscle (with or without hypoaplasia of the overlaying mammary gland/areola/nipple) generates a difference in radiolucency of the lungs at standard X-rays. Such a radiological presentation is in common with a wide variety of acquired disorders and congenital conditions, whose extent is much beyond the scope of these recommendations. It is relevant, however, to note that, in addition to PS, many different pleural, lung parenchymal, pulmonary vasculature, central airways and mediastinal anomalies, as well as technical issues (e.g. patient rotation and lateral decentering) may cause unilateral hyperlucency of the lungs at standard X-rays [20, 21].

**Table 5:** Recommendations for differential diagnoses

		Grade	Consensus agreement
R5.24	In unilateral PS, differential diagnosis includes: (i) congenital or acquired thoracic soft-tissue anomalies (including isolated unilateral mammary gland/areola/nipple hypo/aplasia, localized lipoatrophy, morphea, Parry-Romberg syndrome, Becker nevus syndrome, surgery, traumas); (ii) asymmetry of the thoracic bony structures, due to thoracic scoliosis and/or bony anomalies; (iii) unilateral acquired or congenital defects of the diaphragm.	Possibly useful/modest literature	100%
R5.25	In bilateral PS, differential diagnosis should also consider skeletal dysplasias affecting the rib cages (“thoracic dysplasia”).	Possibly useful/modest literature	77,8%
R5.26	In PS with upper limb anomalies, differential diagnosis should consider ectrodactyly, and, in a minor extent, transverse upper limb defects.	Possibly useful/modest literature	100%
R5.27	In complex phenotypes, it should be considered that PS commonly present with Sprengel deformity and less frequently with Moebius sequence (Poland-Moebius syndrome). Single patients combining PS with Adams-Oliver syndrome, Klippel-Feil sequence, facio-auriculo-vertebral dysplasia and frontonasal dysplasia have been also described. Finally, PS can be also part of the Carey-Fineman-Ziter, a congenital myopathy due to recessive variants in *MYMK*.	Definitely useful/strong literature	91,7%

#### Communicating the diagnosis

The diagnosis of PS is arrived at in most cases within the first year of life [33]. It is often communicated to parents who are unaware of the presence of the disorder [34]. As for any newborn with congenital anomalies the diagnostic pathway and process should be communicated to the parents by explaining that the waiting time is necessary to receive the results of all requested investigations for an accurate evaluation (Table 6). The diagnosis should be addressed to both parents, since they are the “care coordinator”, a role that should be filled formally by a provider of the healthcare system [35]. The information should be delivered gradually and adapted to the family’s socio-cultural level. Once the medical team is confident in the diagnosis of PS, it is important to underline that PS is not progressive and that, in the absence of severe rib cage malformations, its survival rate is comparable with the general population. A normal psychomotor development is expected and the malformation does not affect the growth. Correct education allows the development of manual skills even in the carriers of severe malformations. The communication of the diagnosis should occur in the presence of a psychologist or if this is not possible, an external psychological help should be suggested to support families and patients in coping with the disease. The continuity of care must be guaranteed and, if the specialist surgeon is not available in the clinical team, the contacts of the most appropriate specialist (s) for surgery must be given to the patients/families during the diagnosis and the follow-up. They should be also informed about parent/patient organizations [36] such as AISP. A minority of cases is diagnosed in adolescence or adulthood. Dissatisfaction with body shape can be a source of distress that may significantly affect the dimension of one’s health. Puberty, in particular, is characterized by many sometimes dramatic physical, physiologic, biochemical and personality changes that occur when teens are particularly sensitive to the opinions of others [37].

**Table 6:** Recommendations for communicating the diagnosis

		Grade	Consensus agreement
R6.28	In the communication of the diagnosis it is important to underline that PS is not progressive congenital anomaly and does not associate with abnormal psychomotor development.	Definitely useful/strong literature	92,9%
R6.29	In cases without severe thoracic malformation, the communication of the diagnosis should include a statement on the survival rate which is comparable with the general population.	Definitely useful/strong literature	100%
R6.30	As PS is an exclusion diagnosis, the parents and family should be informed on the need of waiting the results of all requested investigations before fixing the diagnosis.	Definitely useful/strong literature	84,6%
R6.31	The communication of the diagnosis should be made in the presence of a psychologist. If the psychological support is not available at the time of the diagnosis an external psychological should be advised.	Definitely useful/strong literature	90,9%
R6.32	Contacts of the most appropriate specialist(s) for surgery must be given to the patients/families during the diagnosis and the follow-up. They should be also informed about available local, national and international support groups and/or patients associations	Definitely useful/strong literature	92,9%

#### Genetic counseling

Most cases are sporadic, affected children of unaffected parents with no family history of congenital anomalies. Rare familial cases are reported in literature with a rate ranging from 4.2% (PS families with recurrence of pectoral muscle defects) to 8.4% (Poland-like syndrome families with PS index case and ≥ 1 relative(s) showing normal pectoral muscles but with upper limb and/or thoracic anomalies common in PS) [9]. In the presence of sporadic cases the risk of recurrence, i.e. the risk that the same couple has a second affected child, is low. For familial cases, the recurrence risk is higher than that of the general population and must be calculated in accordance to the possible inheritance pattern (Table 7). It is therefore of fundamental importance to clinically evaluate the parents of affected children to exclude they could present mild, underdiagnosed forms that could therefore turn an apparently sporadic case into a familial case thus increasing the risk of recurrence. Genetic counseling is essential in all cases of PS and should be focused on informing patients that:

- the pathogenic mechanisms underlying PS are still unknown;
- most PS patients present in isolated form;
- most cases of PS are sporadic but about less than 10% show familial recurrence, with higher prevalence in males, which suggests a genetic, hereditary basis;
- to date, different models of transmission have been hypothesized, including autosomal recessive (recurrence risk of 25%) and autosomal dominant with incomplete penetrance (up to a 50% risk of recurrence);
- sporadic cases can be explained by the appearance of de novo mutations;
- non-recurrent genomic rearrangements have been occasionally associated with PS [38, 39].

**Table 7:** Recommendations for genetic counseling

		Grade	Consensus agreement
R7.33	At the moment, PS is considered a sporadic disorder. Familial recurrence is really exceptional. Therefore, general counseling for sporadic cases in families with a previously affected child or for affected adults with negative family history should be reassuring concerning the chance of recurrence in a future pregnancy.	Definitely useful/strong literature	100%
R7.34	Genetic counseling in families with multiple affected individuals should prompt the revision of the clinical diagnosis. If the diagnosis is confirmed in multiple affected individuals, genetic counseling should consider specific Mendelian inheritance patterns or, perhaps, multifactorial inheritance.	Definitely useful/strong literature	87,5%
R7.35	Because the molecular basis of PS is mostly unknown a confirmatory molecular test applicable in prenatal diagnosis is not available and genetic counseling remains unsupported by laboratory tests.	Definitely useful/strong literature	100%
R7.36	Cytogenomic, molecular and exomic investigations should be limited to the more complex cases; no routine laboratory investigation is available for confirming the diagnosis of PS.	Possibly useful/modest literature	100%
R7.37	As PS is a congenital disorder, genetic counselling is recommended in all cases	GCP (no literature available)	100%

### Surgical treatments and specific medical therapies on the major symptoms

There is not any definitive solution to treat PS as well as any other genetically determined malformation syndrome. However, a surgical treatment of the major abnormalities and medical complications are appropriate and do not differ from that of other similar isolated malformations in the general population.

#### Thoracic surgery

In the literature, there are no strict guidelines or indications regarding thoracic surgery in PS (Table 8). In the experience of the group of pediatric and thoracic surgeons who are the authors of the present recommendations, thoracic surgery is not always necessary in PS [40]. The indication to thoracic surgery are usually related to severe rib cage anomalies, either regarding anterior chest wall (pectus excavatum, carinatum or combination of both) or ribs (rib agenesis). In case of anterior chest wall defect, the indication to the surgery can be cosmetical or functional. If the heart or the lungs are compressed by a pectus excavatum, a retrosternal metallic bar (Nuss procedure) is helpful to lift the sternum up and releiving the visceral compression. In case of asymmetric anterior chest wall without heart/lung compression, cosmesis can be improved with cartilage resection or osteotomy or both. In young patients, with a compliant chest wall, conservative treatments as FMF brace or vacuum bell can be used. In case of rib agenesis, surgery aims to give more protection to the thoracic organs, although no reports of traumatic complications have been reported in PS patients to the best of our knowledge. Thoracoplasty for rib agenesis is performed also to abolish paradoxycal respiration and stabilize the lungs and rib cage during forced inspiration/exhalation cycle. In order to obtain the stabilization of rib cage, different surgical solutions can be chosen, mainly using non absorbable prosthetic mesh or metallic prosthesis. Goretex mesh can be used successfully especially if the rib defect is not too wide, usually in case of absence of one or two ribs. If the defect involves three or more ribs, it is better to reinforce the rib cage by using a metallic prosthesis. In recent years, 3D printed metallic prostheses have been successfully implanted in PS patients. Goretex mesh is usually used above the metallic prosthesis to avoid skin erosion. It is usually better to correct a severe chest wall anomaly (if present) before breast/pectoral reconstruction, to improve the final result. In some cases, chest wall anomaly can be corrected at the same time of plastic surgery first step. The combined approach has the advantage of optimizing skin incision, reducing the surgical procedures, the number of hospitalizations and the dyscomfort for the patient. Regarding the best age for surgery, it is usually not necessary to operate the patients before 12-13 years of age. Ideally, thoracic reconstruction around 14-15 years of age when the thoracic growth is already advanced is our preferred time.

**Table 8:** Recommendations for thoracic surgery

		Grade	Consensus agreement
R8.38	Respiratory symptoms are not common in PS patients. Lack of protection of lungs and other thoracic organs due to the rib cage defect (rib agenesis) does not indicate per se thoracoplasty during childhood.	Definitely useful/strong literature	57,1%
R8.39	It is better to avoid non resorbable materials before 12 years of age.	Possibly useful/modest literature	80,0%
R8.40	Conservative methods (vacuum bell, FMF or corset for pectus carinatum) are promising tools to treat pectus excavatum and carinatum associated with PS in young patients.	Definitely useful/strong literature	100%
R8.41	The management of pectus excavatum and pectus carinatum should be evaluated for each case and can be carried out through conservative strategies (vacuum bell, braces) or interventional ones (Nuss procedure, surgical treatment of pectus carinatum), although surgery in absence of respiratory symptoms should be postponed at least until the beginning of adolescence, towards the completion of the growth of the thoracic wall	Definitely useful/strong literature	57,1%
R8.42	PS can be classified in minimal (only pectoral defect), partial (thoracic or upper arm variant) and complete form	Definitely useful/strong literature	88,9%
R8.43	TBN classification is useful to classify the thoracic defect in PS	Definitely useful/strong literature	100%
R8.44	Early evaluations of patients optimizes the treatment and is better for psychological reasons.	Possibly useful/modest literature	100%
R8.45	In selected cases, 3D Printing and new technologies can be helpful to build prosteses custom made for thoracic reconstruction in PS	Definitely useful/strong literature	100%
R8.46	Combined surgical treatment (thoracic and plastic surgery) can reduce the number of surgical procedures.	Definitely useful/strong literature	100%

#### Hand surgery

Hand anomalies in PS can show many forms of clinical presentation varying from a hypoplasic but functional hand to severe grade of symbrachydactyly with one or more absent fingers (Table 9). Moreover, there are a group of patients which are not affected by hand malformation, presenting the only absence of pectoral muscles. Clinical examination should not be limited to the hand but the entire upper limb must be checked because of the chance of malformations affecting the wrist, the elbow and the shoulder. Classifying the anomalies of the hand represents an important step which has a direct correlation with the choice of treatment; many classifications have been proposed but the majority were focused exclusively on the hand missing the likelihood of an involvement of other district of the upper limb. In 2012, a new system has been published and it can be considered more useful because it takes into consideration the entire upper limb [5]. Eight types of clinical aspects have been considered:

I: Absence of hand/upper limb anomalies
II: Hypoplastic hand without morphologic and functional anomalies
III: Symbrachydactyly with 5 functional fingers and possible morphologic anomalies of the phalanges and partial range of motion
IV: Symbrachydactyly with some functional fingers
V: Symbrachydactyly with absent or nonfunctioning fingers
VI: Classic hand anomalies of PS with proximal radioulnar synostosis
VII: Classic hand anomalies of PS with congenital high scapula
VIII: Other associated anomalies

**Table 9:** Recommendations for hand surgery

		Grade	Consensus agreement
R9.47	It is mandatory to check all the upper limb in order to identify any malformations (shoulder, elbow) associated with hand anomalies	Definitely useful/strong literature	100%
R9.48	Reconstructive planning should be adapted to the type of deformity of the hand	Definitely useful/strong literature	100%
R9.49	The correction of syndactyly should begin between 12 and 24 months of life; if the first web space is involved, surgery should be performed between 6 and 12 months.	Definitely useful/strong literature	100%
R9.50	If phalanx are absent, two options should be proposed to the parents: microvascular digital transfer from the foot or non-microvascular free phalangeal transfer from the foot	Definitely useful/strong literature	100%
R9.51	The patient must be followed until the end of skeletal growth because recurrence of syndactyly, secondary to scar hypetrophy, may be possible	Definitely useful/strong literature	100%
R9.52	When a recurrence occurs, it should be corrected during adolescence in order to reach a definitive result	Definitely useful/strong literature	100%
R9.53	We recommend to use the following classification (useful for treatment therapeutic of hand function) of Hand and Upper Limb anomalies in PS: I Absence of hand/upper limb anomalies II Hypoplastic hand without morphologic and functional anomalies III Symbrachydactyly with 5 functional fingers and possible morphologic anomalies of the phalanges and partial range of motion (ROM) IV Symbrachydactyly with some functional fingers V Symbrachydactyly with absent or nonfunctioning fingers VI Classic hand anomalies of PS with proximal radioulnar synostosis VII Classic hand anomalies of PS with congenital high scapula VIII Other associated anomalies	Definitely useful/strong literature	100%
R9.54	Types I and II (R2.11) do not need any surgical treatments, which, however, is necessary for type III and, in particular, for types IV and V, to improve hand function	Definitely useful/strong literature	100%
R9.55	The reconstruction of the second and third webspaces can be delayed until 18 months of age without adverse effect on hand function or fine motor development	Definitely useful/strong literature	100%
R9.56	Early surgery is recommended for border digits as syndactyly between digits of disparate length may result in flexion contracture or angular deformity.	Definitely useful/strong literature	100%
R9.57	Minor syndactyly, such as observed in PS, can be treated by the usual methods of local enlargement plasty of the first commissure: trident plasty (YV double Z), Z plasty at four tatters	Definitely useful/strong literature	100%
R9.58	The use HA scaffold for skin regeneration in syndactyly release surgery in young children represent a valid alternative to the use of skin grafts	Definitely useful/strong literature	100%
R9.59	The first wound care is recommended after 3 weeks post-surgery	Definitely useful/strong literature	66,7%

Reconstructive planning should be discussed with the parents, adapting it to the type of deformity.

Children with normal hand (type I) or with hypoplasic but functional hand (type II) do not need any surgical treatment which is necessary instead for type III and in particular for types IV and V in order the improve hand function.

The issues the surgeon must deal with are the presence of syndactyly and the absence of phalanges (typically the middle phalanx) or entire fingers. Timing of surgery depends on the type of malformation. The correction of syndactyly should begin between 12 and 24 months of life, however, in case of syndactyly between finger of disparate length, early surgery is recommend between 6 and 12 months in order to reduce the risk of flexion contracture or angular deformities [41]. The reconstruction of the second and third web spaces can be delayed until 18 months of age without adverse effect on hand function development. Surgical techniques do not differ from those adopted in other forms of syndactyly by means of local flaps (VY – Double Z – Z plasty at four tatters) to reconstruction the web space as well as the digital surfaces. Surgeons may need to use a skin graft for the uncovered areas; the volar surface of the wrist or the groin are the most common donor site. However, the use of hyaluronic acid scaffold for skin regeneration in syndactyly release surgery may be take into consideration as an alternative to the use of skin graft, due to the good results reported in young patients [42]. After surgery an occlusive bandage should be applied and the first wound care is recommended after 2 or 3 weeks.

However, some patient may need an early dressing in case of infection, exudate or insufficient care of the dressing by the parents. In case of symbrachydactyly with phalanx absence, two options may be proposed to the parents, according to the grade of malformation: i) Non microvascular free phalangeal transfer from the foot, or 22) Microvascular digital transfer from the foot.

The first option should be proposed in case of symbrachydactyly with the presence of the proximal phalanx in order to increase the length of the single ray. Conversely, microvascular transfer is the only option in case of a peromelic hand or in the rare situation of a congenital metacarpal amputation. However, parents are often frightened from the idea of using the foot as donor site as well as from the risk of failure of a microsurgical transfer whereas they usually accept better the proposal of a free phalangeal transfer also considering the lesser functional and aesthetic result.

Finally, physicians must follow the patient until the end of skeletal of skeletal growth, because recurrence of syndactyly, secondary to scar hypertrophy, may be possible. When a recurrence occurs, it should be corrected, when possible, during adolescence in order to reach a definitive result.

#### Plastic surgery

Reconstructive surgery in PS has a mainly aesthetic meaning and it aims to improve quality of life (Table 10). The asymmetry involving the breasts, in fact, is often moderate or severe and it is made even more complex by the underlying thoracic malformations. Social aspects in case of bodily malformation cannot be ignored, both for male and female patients. A single defect, in fact, can affect the overall physical appearance up to create a variable degree of uneasiness in social relations [43]. Patients affected by PS seem to experience high level of body uneasiness during adolescence [37]. The reconstructive path, for this reason, can start during puberty and it depends strictly on the characteristics of the mammary / thoracic malformation, on the anatomical characteristics of the patient and on the psychological condition linked to the malformation. First of all it is important to classify the malformation: TNB classification is the most recent specific classification of thoracic, breast and nipple malformations which guides the surgeon in choosing the surgical path [40, 44]. A customized approach is therefore necessary even in relation to patient’s age, expectations and needs [43]. Patients suffering of PS can contact the surgeon at any age depending on the time of diagnosis. Specialist interviews in the neonatal period and/or before puberty are only intended to reassure caregivers. Reconstructive plan can benefit from the modern breast reconstructive surgery techniques and should be the least invasive and debilitating for the patient given the main aesthetic purpose of the reconstruction and the musculoskeletal malformative characteristics that often affect the chest. Muscle transpositions should be used in selected cases after an accurate evaluation of the pros and cons of the procedure based on the anatomical characteristics (back, shoulder, posture, …) and life habits (sport activity, work activity, hobbies, …) of the patient. Muscle transpositions shouldn’t be used in non-adult patients, or even in patients who have not fully completed their psycho-physical development and who have not clearly outlined their social and working life habits [33]. Reconstructive surgery can help especially younger patients (teenagers) to experience the aesthetic stigmata of the disease less traumatically, accompanying them on a path of growth and positive development of their body image. Soft tissue reconstructive surgery cannot be definitive; breast, muscles and adipose tissue involved in the malformative feature are tissues in continuous evolution based on age, physiological changes (pregnancy) and life habits and, for this reason, it may be necessary to rework them over time to maintain the desired aesthetic result.

**Table 10:** Recommendations for plastic surgery

		Grade	Consensus agreement
R10.60	Reconstructive plan should be the least invasive and debilitating for the patient given the main aesthetic purpose of the reconstruction	Definitely useful/strong literature	90,0%
R10.61	Autologous fat graft should be the first surgical procedure but it is strictly dependent to the grade of deformities, BMI index and chest wall involvement.	Definitely useful/strong literature	62,5%
R10.62	Breast implants are the simplest solution to obtain missing breast volumes	Definitely useful/strong literature	100%
R10.63	The contralateral breast should be reworked as little as possible, especially in young nulliparous patients	Possibly useful/modest literature	80%
R10.64	Skin expansion should be planned if side-affected nipple dislocation exceeds 2 cm (N2 in TNB classification) or if the side-affected breast is absent (N3,B2 in TNB classification).	Definitely useful/strong literature	75%
R10.65	Muscle transpositions should be used in strictly selected cases after an accurate evaluation of the pros and cons of the procedure based on the anatomical characteristics (back, shoulder, posture, …) and life habits (sport activity, work activity, hobbies, …) of the patient.	Possibly useful/modest literature	100%
R10.66	Muscle transpositions shouldn’t be used in non-adult patients, or even in patients who have not fully completed their psycho-physical development and who have not clearly outlined their social and working life habits.	Definitely useful/strong literature	100%

The rehabilitation plan must be scheduled after each intervention on the basis of illness-specific criteria and specific surgical procedures (Table 11).

**Table 11:** Recommendations for physical therapies

		Grade	Consensus agreement
R11.67	It’s necessary to monitor vertebral column and thoracic symmetry during the growth	Definitely useful/strong literature	100%
R11.68	To evaluate scapulo-thoraco-humeral dynamics is recommended	Possibly useful/modest literature	100%
R11.69	To evaluate symmetry of development of the upper limb muscular masses is recommended	Possibly useful/modest literature	100%
R11.70	Pysiatrist visit to assess the feasibility of a reconstructive intervention of transposition of the gran dorsal muscle is recommended	GCP (no literature available)	66,7%
R11.72	To evaluate use of upper limb gestures is recommended	GCP (no literature available)	100%
R11.73	The following evaluations to correct aesthetic/functional balance are highly recommended and must be supportive in surgery decision: a) to monitor the step of psycho-motor development in the upper limb use, b) to evaluate the active and passive range of motion if are different, c) to evaluate the single prehensile movements age-related and to observe the preferred patterns of usage, d) to measure the pinch and grip strength	Possibly useful/modest literature	100%

### Psychological issues and social assistance

Currently, there is no scientific literature on psychological support for patients, and their families, suffering from PS (Table 12). In contrast, clinical practice highlights the importance of psychological support for the patient and his family. Furthermore, the psychologist appears to be an important resource also within the multidisciplinary specialist team both for drafting the protocols for taking charge [45] and for supporting colleagues during the various stages of the diagnostic, care and assistance process [46]. Since the syndrome is increasingly diagnosed at birth, psychological support must first be directed to parents to elaborate painful experiences that could affect the attachment and care of the child and thus reinforce parenting skills. Subsequently, as the child reaches more and more autonomy, the support is necessary both for the parents and for the subject himself to avoid developing feelings of inadequacy and consequent socialization problems. About the choice to undergo surgery, especially in adolescence, it is useful that the decision is reached with the help of the psychologist, after assessing the subject’s psychological conditions, in full awareness and self-determination so that the result is satisfactory [47]. It is not recommended to take the intervention exclusively on the advice of medical specialists. When the diagnosis is late, in adolescent or adult age, the psychodiagnostic evaluation is strongly recommended to check how the anomalies caused by the syndrome have affected the subject’s life [48], self-image and relationships with others [49]. The request for correction of anomalies must be carefully considered and examined in depth to exclude unrealistic and unrealizable expectations [50].

**Table 12:** Recommendations for psychological support

		Grade	Consensus agreement
R12.74	To perform a psychological evaluation before facing the reconstructive intervention related to thoracic and/or mammary deformity	GCP (no literature available)	100%
R12.75	Psychological support is fundamental since the diagnosis of the PS to avoid the neurosis onset	GCP (no literature available)	100%
R12.76	To elaborate the diagnosis and to reinforce parental capacities	GCP (no literature available)	100%
R12.77	We recommend a preliminary assessment of psychological condition in adolescent patients, to guide self- consciousness and knowledge of individual needs in order to reach self -determination about surgical operation	GCP (no literature available)	100%
R12.78	We recommend previous assessment of psychological condition in adult patients, to help on accepting the condition and to face up the associate consequences, including surgical treatment or therapy	GCP (no literature available)	100%
R12.79	The psychologist is an important resource for the entire multidisciplinary team during the diagnostic, care and assistance process, helping to build the best path for the specific situation (for example, for the communication of the diagnosis)	Definitely useful/strong literature	100%

### Clinical follow-up and general management

Following the diagnostic classification, people affected with PS are followed by periodic follow-up visits to manage the main complications of the disease, such as functional and aesthetic issues (abnormalities of limbs, paroxysmal movements of the chest wall, reduced lung function, muscle weakness, scoliosis). Pediatric and plastic surgery examination for thoracic abnormalities (ribs, condrosternal cartilages, pectoral muscles, mammary gland) involves periodic clinical examinations and surgical correction in the most complex cases (Table 13).

**Table 13:** Recommendations for clinical follow-up

		Grade	Consensus agreement
R13.80	Thoracic surgery in pediatric age has to be planned and performed by pediatric surgeons.	Definitely useful/strong literature	100%
R13.81	The role of the pediatric thoracic surgeon includes: evaluation of thoracic symmetry, assessment of ribs anomalies, evaluation of the sternum, long-term follow-up, possible surgical treatment.	Definitely useful/strong literature	100%
R13.82	The following evaluations have to be planned for each newly diagnosed patient < 18 yrs. of age: Pediatric thoracic surgeon clinical evaluation, Chest x-ray, Cardiac evaluation with cardiac US, Genetic counseling, Orthopedic evaluation, Plastic surgeon evaluation for pre-adolescents and teenagers, Abdominal ultrasound.	Definitely useful/strong literature	87,5%
R13.83	We recommend for child patients a preliminary assessment of psychological condition, to guide the adequate development of body image and to prevent inferiority feelings.	GCP (no literature available)	100%

The orthopedic evaluation and the hand surgery visit are indicated for the management of postural and upper limb anomalies, with a focus in the hand function. Periodical follow-up and physiotherapy in the most complex cases are also recommended (Table 14). Since in (rare) PS cases are also described for the presence of genitourinary (renal agenesis, renal ectopia, vesico-ureteral reflux and cryptorchidism) and cardiac (dextrocardia and inter-atrial defect) malformations it is advised to perform relevant specialist assessments when appropriate. Regarding risks and potential complications such as breast implant-associated anaplastic large cell lymphoma (BIA-ALCL) in case of breast reconstruction with textured breast implant device, periodic clinical examination is recommended, as one would do to screen for implant complications such as capsular contracture.

**Table 14:** Recommendations for clinical follow-up

		Grade	Consensus agreement
R14.84	Annual follow-up in case of surgery, especially in case of reconstruction with breast or pectoral implant (medical examination, ultrasound) is recommended	Definitely useful/strong literature	100%
R14.85	Provide adequate explanations about the need to perform more or less invasive surgical adjustments to maintain the symmetry between the two hemilates in relation to the physiological changes of the body	GCP (no literature available)	83,3%
R14.86	The patient with PS need to be assisted by a multidisciplinary team (coordinated by a Case Manager) tailored on the basis of the real needs of the patient/family. In general the team should involve the following specialists: Pediatric/thoracic surgeon, Plastic surgeon, Orthopedic surgeon, Hand surgeon, Radiologist, Geneticist, Psychologist, Cardiologist, Ophthalmologist, and other professionals as needed	Definitely useful/strong literature	100%
R14.87	A strong relationship with Patients Advocacy Organizations, both national and international ones, is crucial for the best care of patients with PS	Definitely useful/strong literature	100%
R14.88	If there is not functional limitations there is no need of surgery but patients could decide to undergo surgery for aesthetic reasons.	Definitely useful/strong literature	70%

### Final remarks

The development of best standards of care and commonly approved procedures is urgently needed for all rare diseases (www.rarebestpractices.eu). In fact, to advance knowledge of PS the primary goal is defining evidence-based guidelines for those affected and their caregivers (Table 15). The present working group has formulated a total of 91 recommendations for the diagnosis and management of PS, based on a systematic review of literature and a consensus procedure. In total, 69 recommendations within 14 general principles, ranging from diagnosis to therapeutic approaches have been accepted with > 90% agreement among experts. When summarizing the degree of evidences available for the recommendations proposed in this review, some are “Good Clinical Practice” based on clinical experience of the authors. Key topics include assessment of the rib cage and other associated skeletal anomalies or abnormalities. Careful monitoring of the status and well-being of patients by an experienced multidisciplinary team and validated scores for the progression and follow-up are required in order to perform a structured assessment of the outcomes, as are long-term follow-up studies to clarify the risks of complications of therapeutic approaches. Given the rarity of PS, international collaboration is essential to provide specific support even in geographic areas lacking expert specialists.

**Table 15:** General recommendations

		Grade	Consensus agreement
R15.89	Our knowledge of epidemiology of PS should be improved	Definitely useful/strong literature	100%
R15.90	The precise cause of PS is not known yet: further studies are urgent to find the reasons why PS occurs but research should focus into the etiopathogenesis of PS	Definitely useful/strong literature	100%
R15.91	Standardization of protocols on a national and international basis is needed	Definitely useful/strong literature	100%

## Conclusions

To conclude, this initiative is based on the personal opinion of experts based on the best available evidence and provides recommendations for the options of diagnosis and treatment of PS patients, in order to improve the outcome for those affected. It will now be important to extend the discussion and the acceptability of our recommendations to a wider community of clinicians and families: a large international meeting on PS is expected to be scheduled in 2021 under the direct management of the AISP. In fact, one of the main purposes of the AISP is to improve the care and follow-up of people living with PS. For this reason the AISP has recently developed, in close collaboration with some clinical centers for the treatment of PS in Italy, the first registry for PS available in the world. The registry of PS is a tool for the systematic collection of personal and phenotypic data of affected people. Through the digitalization of high-quality clinical data, the registry aims to improve knowledge of PS and maximize natural history and epidemiologic studies.

### The point of view of patients and caregivers

Purpose of AISP has always been to enhance the network of specialists and medical facilities who collaborates each other in order to allow the best possible care for patients on the Italian territory. Thanks to the drafting of this document, AISP did one important step closer to reach this aim and hope to provide clinicians and patients diagnostic and therapeutic approaches in PS as uniform as possible. AISP will surely advertise this report on its website and its social channels. This document will be shared with other European associations of PS patients and rare disease associations in Italy and in Europe and with the ERN of rare diseases. With the help of the clinicians involved in the draft, the document will be distributed to scientific society, medical structures, general practitioners and pediatricians, asking them to collaborate in its sharing. Moreover an informative brochure will be produced in order to enable patients to fully understand the document. The update of these recommendations will be promoted by AISP as part of its constant scientific activity and will be an integral part of the scientific program of AISP National Conferences.

## Data Availability

Data sharing not applicable as no datasets were generated during the current study.

## Abbreviations

PS: Poland Syndrome
AISP: Associazione Italiana Sindrome di Poland
GCP: Good Clinical Practice
FMF: Freire Martinez Ferro
TBN: Thoracic/Breast/Nipple-areola complex
ERN: European References Network
MRI: Magnetic Resonance Imaging
CT: Computed tomography
